# Sarcoidosis Occurring After Lymphoma: Report of 14 Patients and Review of the Literature

**DOI:** 10.1097/MD.0000000000000121

**Published:** 2014-10-31

**Authors:** Jonathan London, Aurélie Grados, Christophe Fermé, Alexandre Charmillon, François Maurier, Bénédicte Deau, Etienne Crickx, Pauline Brice, Catherine Chapelon-Abric, Corinne Haioun, Barbara Burroni, Marco Alifano, Claire Le Jeunne, Loïc Guillevin, Nathalie Costedoat-Chalumeau, Nicolas Schleinitz, Luc Mouthon, Benjamin Terrier

**Affiliations:** Department of Internal Medicine (JL, CLL, LG, NC-C, LM, BT), National Referral Center for Rare Systemic and Autoimmune Diseases; Department of Hematology (BD), Cochin Hospital, Assistance Publique-Hôpitaux de Paris (AP-HP), Université Paris Descartes; Department of Hematology (EC); Department of Internal Medicine (CC-A), Pitié-Salpêtrière Hospital, AP-HP; Department of Onco-Hematology (PB), Saint-Louis Hospital, AP-HP; Department of Pathology (BB), Hôtel-Dieu Hospital, AP-HP; Department of Thoracic Surgery (MA), Cochin Hospital, AP-HP, Paris; Department of Internal Medicine (AC, FM), Belle Isle Hospital, Metz; Department of Internal Medicine (AG, NS), CHU Conception, Assistance Publique-Hôpitaux de Marseille, Marseille; Department of Medicine (CF), Gustave Roussy, Villejuif; and Lymphoid Malignancies Unit (CH), Henri Mondor Hospital, AP-HP, Créteil, France.

## Abstract

Sarcoidosis is a granulomatous disease that most frequently affects the lungs with pulmonary infiltrates and/or bilateral hilar and mediastinal lymphadenopathy. An association of sarcoidosis and lymphoproliferative disease has previously been reported as the sarcoidosis-lymphoma syndrome. Although this syndrome is characterized by sarcoidosis preceding lymphoma, very few cases of sarcoidosis following lymphoma have been reported. We describe the clinical, biological, and radiological characteristics and outcome of 39 patients presenting with sarcoidosis following lymphoproliferative disease, including 14 previously unreported cases and 25 additional patients, after performing a literature review. Hodgkin lymphoma and non-Hodgkin lymphoma were equally represented. The median delay between lymphoma and sarcoidosis was 18 months. Only 16 patients (41%) required treatment. Sarcoidosis was of mild intensity or self-healing in most cases, and overall clinical response to sarcoidosis was excellent with complete clinical response in 91% of patients. Sarcoidosis was identified after a follow-up computerized tomography scan (CT-scan) or ^18^fluorodeoxyglucose-positron emission tomography/computerized tomography (^18^FDG-PET/CT) evaluation in 18/34 patients (53%). Sarcoidosis is therefore a differential diagnosis to consider when lymphoma relapse is suspected on a CT-scan or ^18^FDG-PET/CT, emphasizing the necessity to rely on histological confirmation of lymphoma relapse.

## INTRODUCTION

Sarcoidosis is a multisystem disease of unknown origin, characterized by the presence of noncaseating epithelioid cell granulomas. It frequently presents in young and middle-aged adults with pulmonary infiltrates and with bilateral hilar and mediastinal lymphadenopathy, and more rarely the eye, the liver, the spleen, the kidneys, and the heart.^1^ The diagnosis of sarcoidosis is based on criteria from the American Thoracic Society (ATS), the European Respiratory Society (ERS), and the World Association of Sarcoidosis and Other Granulomatous Disorders (WASOG).^2^ These criteria include the presence of clinicoradiological findings suggestive of sarcoidosis, the presence of histological evidence of noncaseating epithelioid cell granulomas, and the exclusion of known causes of granulomatous reactions.

The association of sarcoidosis and lymphoma is well established and was named the sarcoidosis-lymphoma syndrome by Brincker^3^ in 1986. In this syndrome, lymphoma occurs after sarcoidosis, and patients often have a chronic active form of sarcoidosis, suggesting that chronic disease could be a risk factor for lymphoma. This finding is supported by the demonstration of an increased risk of Hodgkin lymphoma (HL) of 14.1% in patients with sarcoidosis in a population-based case–control study in Scandinavia.^4^

In addition, granulomatous reactions are frequently associated with lymphoma. Such sarcoid-like reactions have been reported to occur in up to 13.8% of patients with HL and 7.3% of patients with non-Hodgkin lymphomas (NHL).^5–7^ The granulomatous reaction is most often seen in the same lymph node or organ as the lymphoma but can be found in other hematopoietic organs. Sarcoid-like reactions can sometimes be the predominant histological finding with few tumor cells, especially in HL or T-cell lymphomas, making diagnosis of lymphoproliferative disorder difficult.

The sarcoidosis-lymphoma syndrome is a well characterized association, but the onset of sarcoidosis after the lymphoma has only rarely been reported in the literature.^3,8–28^ It is not known whether the association is fortuitous or not.

In the present study, we describe the clinical, biological, and radiological characteristics and the outcome of patients presenting with sarcoidosis occurring after lymphoma. We report the largest published series so far of 14 cases and review the literature.

## PATIENTS AND METHODS

### Patients

We conducted a French national retrospective study in hematology and internal medicine departments. Patients were included if they were >18 years of age and diagnosed with HL or NHL that preceded the onset of sarcoidosis. The diagnosis of sarcoidosis was defined by the association of clinical and radiological findings suggestive of sarcoidosis with histological confirmation, according to the ATS/ERS/WASOG criteria.^2^ Histological samples were obtained from tissue biopsies of lymph nodes (n = 12), bone marrow (n = 1), lung (n = 1), bronchial wall (n = 1), skin (n = 1), or minor salivary glands (n = 2), and characterized by noncaseating epithelioid cell granulomatous lesions without evidence of mycobacterial infection. Other specific granulomatous disorders were excluded. Serum protein electrophoresis was systematically performed to exclude common variable immunodeficiency (CVID). Patients with concomitant sarcoidosis and lymphoma or with evidence of granulomatosis on tissue biopsy at lymphoma onset were also excluded. Diagnosis of lymphoproliferative disorder was based on the World Health Organization classification criteria.^29^ All lymphoproliferative disorders were confirmed by tissue biopsy. A total of 14 patients with sarcoidosis occurring after lymphoma were included. This survey was conducted in compliance with the protocol of Good Clinical Practices and the Declaration of Helsinki principles.

### Clinical and Laboratory Assessment

Clinical and biological data were retrieved for each patient at the time of the lymphoma diagnosis, diagnosis of sarcoidosis, during follow-up, and at the end of follow-up, by J.L. and the practitioners in charge of the patients using a standardized form. Collected data included sex, age at diagnosis of lymphoma and sarcoidosis, lymphoma characteristics, treatment received for lymphoma and outcome, characteristics of sarcoidosis at diagnosis and during follow-up, and treatment received for sarcoidosis and outcome. Imaging findings and ^18^fluorodeoxyglucose-positron emission tomography/computerized tomography (^18^FDG-PET/CT) characteristics of lymphoma and sarcoidosis were recorded when available. Laboratory assessment included serum electrophoresis and immunofixation, serum creatinine level, lactate dehydrogenase (LDH), calcium, angiotensin-converting enzyme (ACE), albumin, C-reactive protein, and complete blood count.

### Outcome and Response to Therapy

Hematological response after treatment of lymphoma was defined as complete response (CR), partial response (PR), and relapse according to the proposed International Workshop Criteria (Cheson criteria).^30^

Regarding sarcoidosis, clinical complete response (CR) was defined by a complete regression of clinical symptoms and clinical PR by a decrease ≥50% of the initial symptoms intensity, without onset of new clinical manifestations. Radiological CR was characterized by normalization of the ^18^FDG-PET/CT or of the CT-scan, and radiological PR by a ≥50% reduction in the size of the initial lesions or lymph nodes and the absence of new radiological findings related to sarcoidosis.

### Review of the Literature

We systematically reviewed the literature for reports of characteristics and clinical outcomes of patients with sarcoidosis occurring after lymphoma diagnosis.

We searched MEDLINE via PubMed for all articles published in English or French using the keywords or MeSH terms “sarcoidosis,” “lymphoma,” “non-Hodgkin lymphoma,” “Hodgkin,” “granulomatous reaction,” and “granuloma.” We reviewed bibliographies of articles retrieved for other relevant articles. Only reports with available clinical, biological, radiological, and histological data and outcomes were included. Reports in which the order of occurrence between sarcoidosis and lymphoproliferative disorder was unknown were excluded. Relevant data were recorded using the same standardized form as for our personal series.

### Statistical Analysis

Descriptive statistics included the mean (SD) or median (minimum–maximum) as appropriate for continuous variables and frequency (percentage) for categorical variables.

## RESULTS

### Characteristics of Patients

We identified 17 patients from 7 different centers who were eligible for the study. Diagnosis of sarcoidosis was made between October 2006 and October 2013. All patients had a history of lymphoma prior to sarcoidosis. Fourteen patients fulfilled the inclusion and exclusion criteria, whereas 3 patients were excluded (1 patient received interferon therapy for melanoma prior to sarcoidosis onset, 1 patient had peripheral neuropathy as the only clinical symptom finally attributed to cryoglobulinemia complicating lymphoplasmacytic lymphoma, and 1 patient who had a history of tuberculosis and necrosis accompanying granuloma in the bone marrow biopsy).

Review of the literature identified 21 studies, including 36 patients with sarcoidosis occurring after lymphoma diagnosis. Eleven patients were excluded for lack of data,^3,8,17,18,22,24,28^ and the 25 remaining patients were included. Combining our personal series and the literature review, 39 patients were analyzed.

The characteristics of the 14 cases from our study and the 25 patients from the literature are shown in Tables 1  and Tables 2  , respectively.

**TABLE 1:**
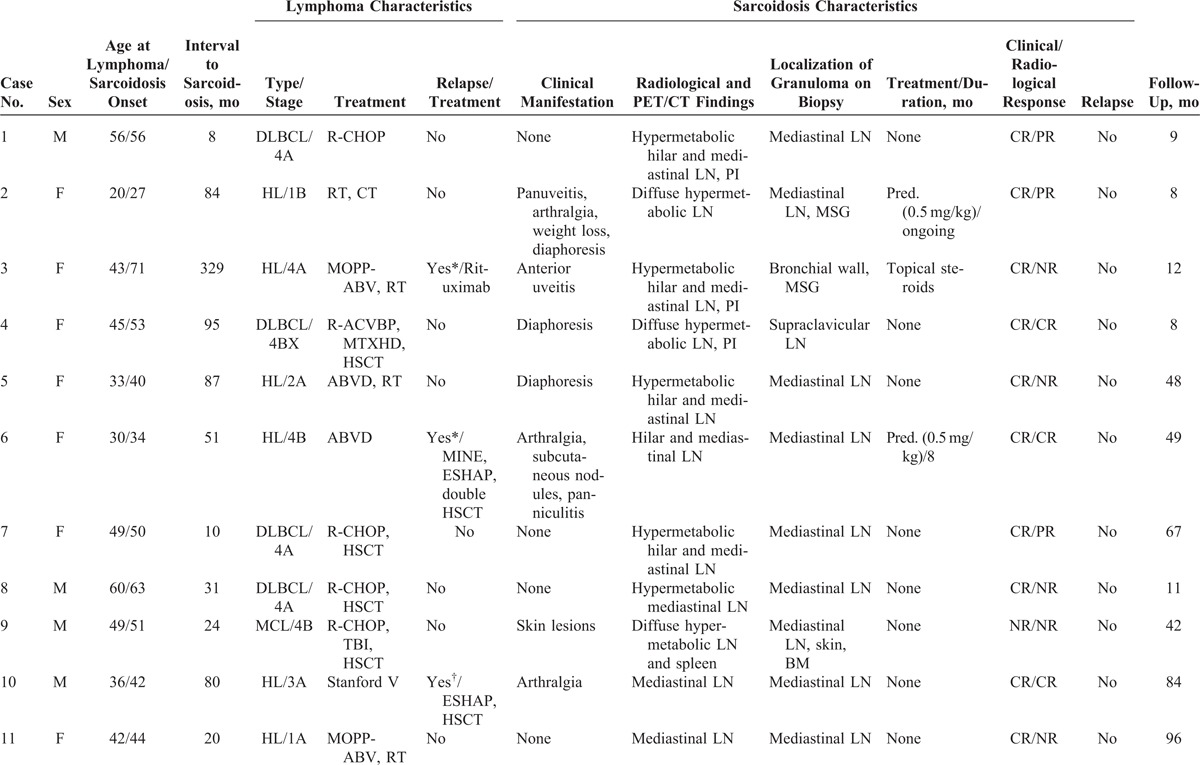
Characteristics of Our 14 Patients

**TABLE 1 (Continued):**
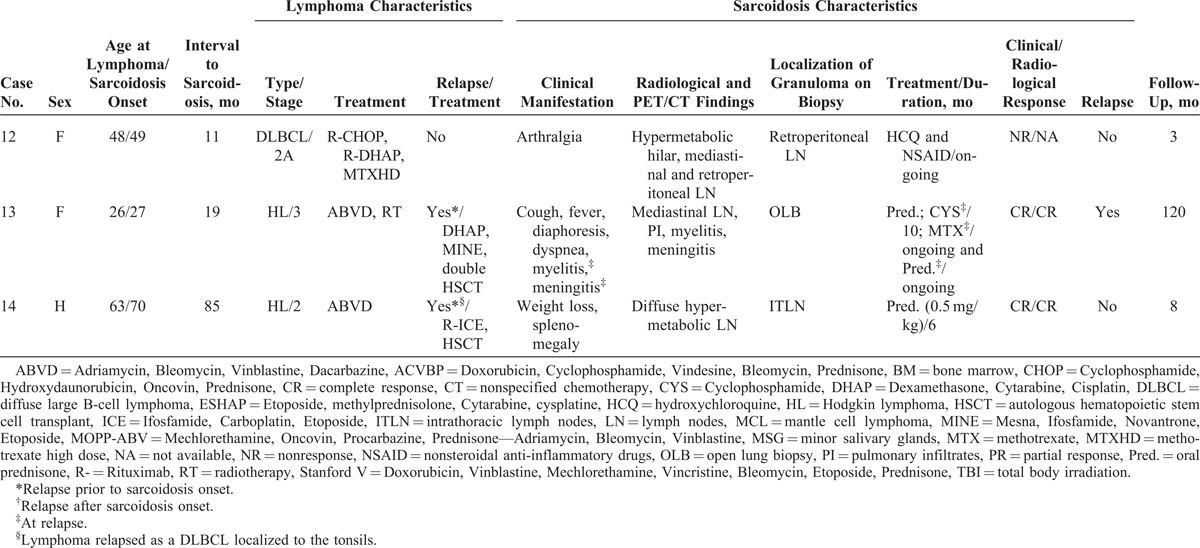
Characteristics of Our 14 Patients

**TABLE 2:**
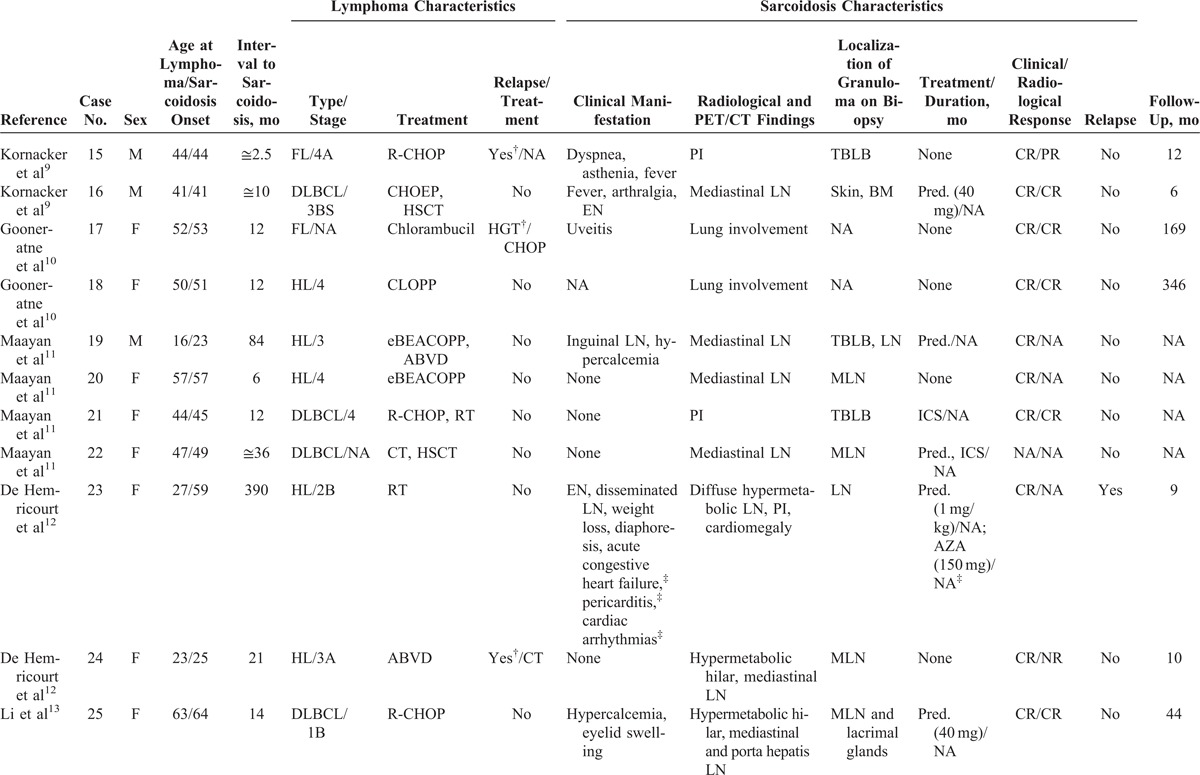
Characteristics of Patients From the Literature Review

**TABLE 2 (Continued):**
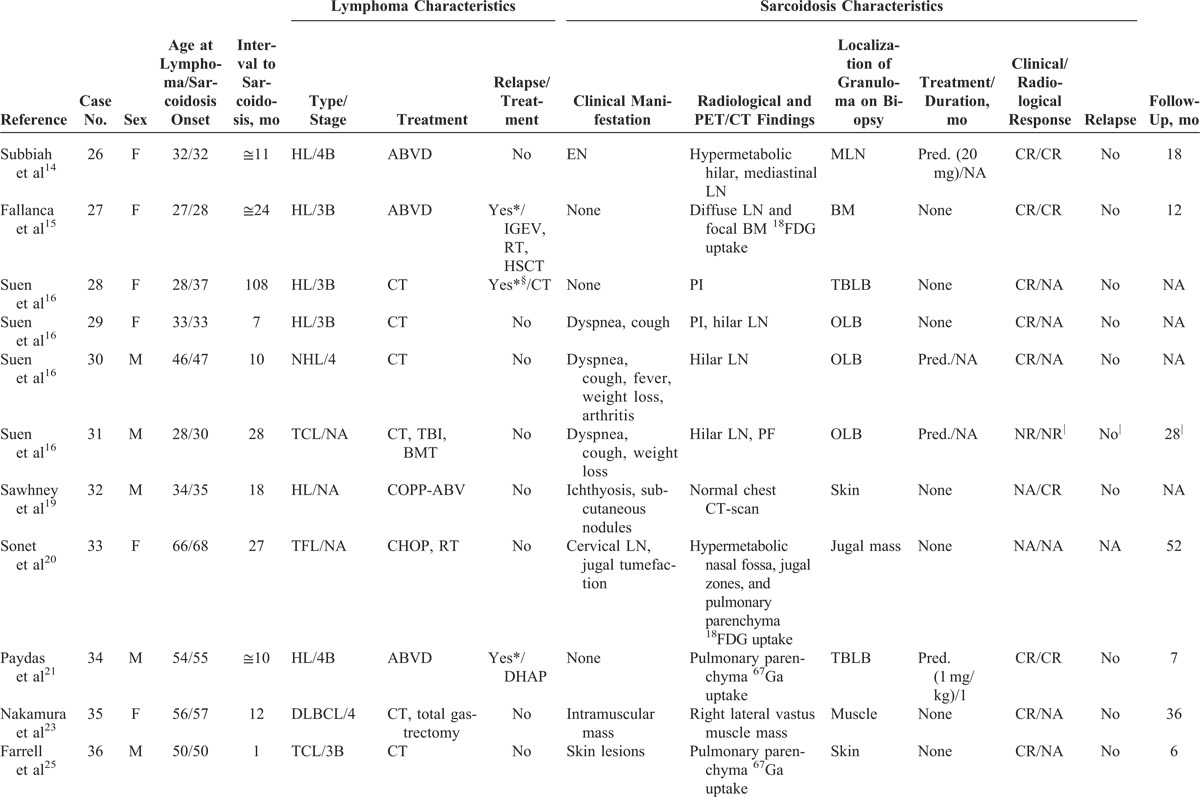
Characteristics of Patients From the Literature Review

**TABLE 2 (Continued):**
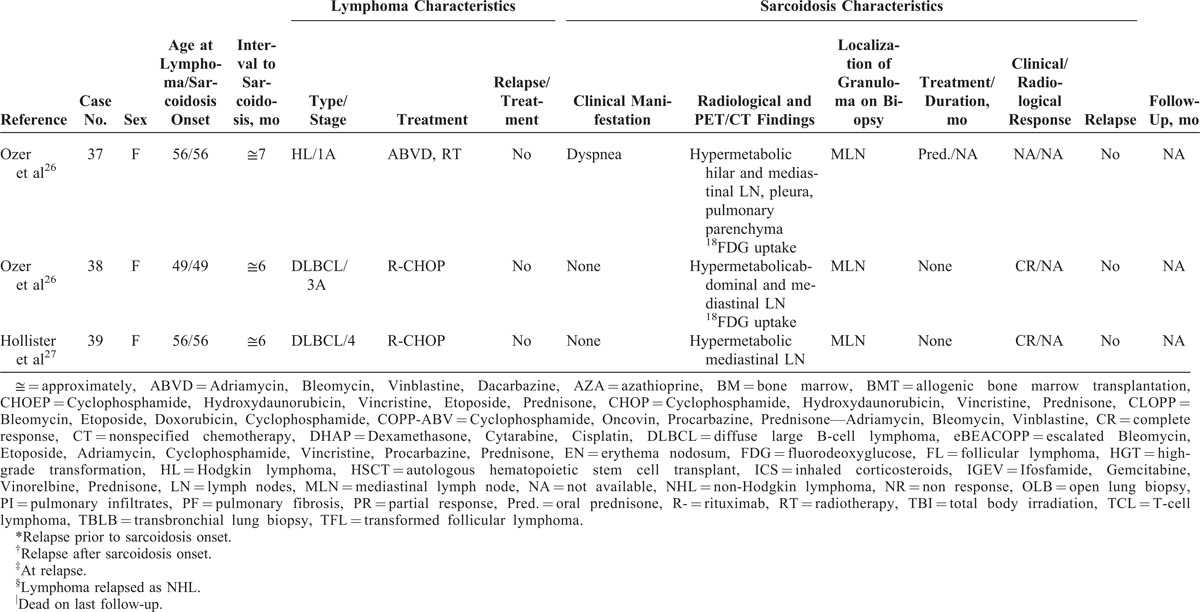
Characteristics of Patients From the Literature Review

### Characteristics of Lymphoma

**FIGURE 1:**
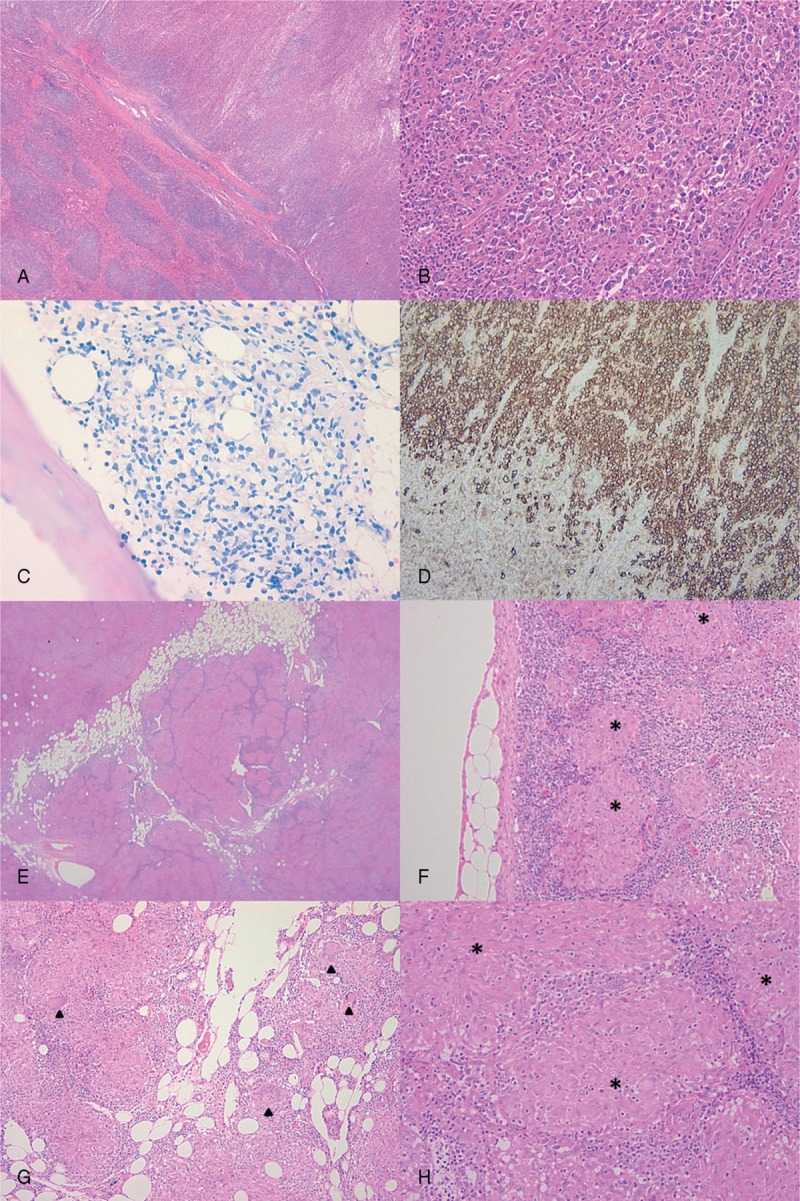
Histological characteristics of lymphoma and sarcoidosis. Spleen (A, B, and D) and bone marrow biopsy (C) of patient 1 showing DLBCL. Microscopic analysis of splenic nodules revealed diffuse large irregular lymphoid cells (A and B, HES ×2 and ×20, respectively). Bone marrow biopsy showed an infiltration of irregular lymphoid cells with the same phenotype as the lymphomatous cells in the spleen (C, Giemsa ×20). CD20 immunostaining confirmed that lymphomatous cells in the spleen were B cells (D, ×10). Mediastinal lymph node biopsy realized 8 month later (E, HES ×2; F and G, HES ×10; H, HES ×20) displayed characteristic features of sarcoid granuloma: noncaseating small granulomatous lesions (stars), consisting of epithelioid cells without evidence of mycobacterial infection. Several giant multinucleated cells are shown on panel G (arrowheads). DLBCL = diffuse large B-cell lymphoma.

Main characteristics of lymphoma diagnosis, treatment, and outcome are detailed in Tables 1  and Tables 2   and summarized in Table 3. Median age at lymphoma diagnosis was 45 years (range, 16–66 years). Twenty patients (51%) had HL and 21 patients (54%) had NHL, with 2 patients having both HL and NHL prior to sarcoidosis. Among patients with NHL, 12 had diffuse large B-cell lymphoma (DLBCL), 3 had follicular lymphoma including 1 transformed follicular lymphoma, 2 had T-cell lymphoma, 1 had mantle cell lymphoma, and 1 patient had NHL without any additional specifications reported. All patients had biopsy-proven lymphoma and none had associated granulomatous reactions on histological examination or had clinical evidence of sarcoidosis (Figure 1).

**TABLE 3:**
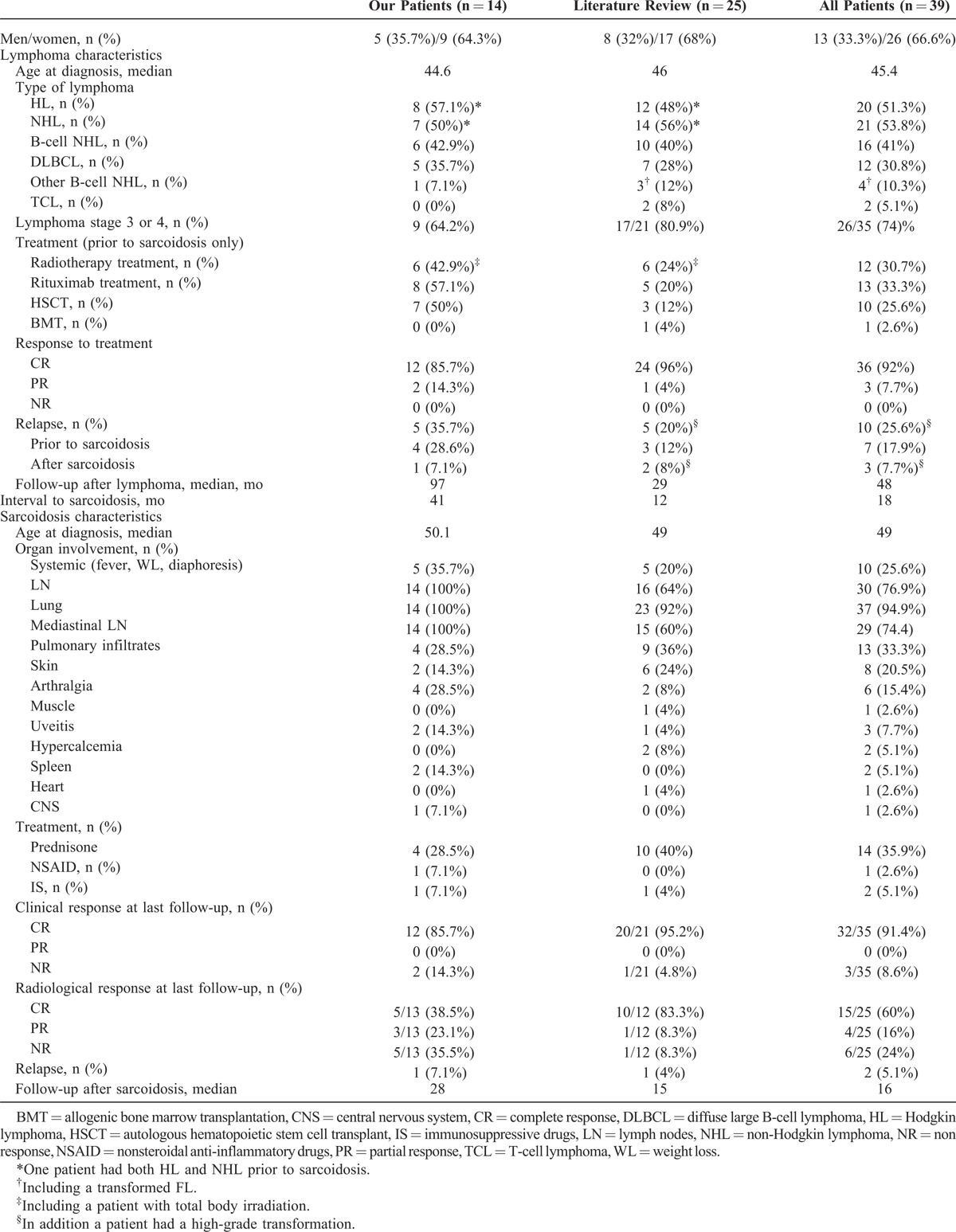
Summary of Patient Characteristics From Our 14 Patients and From the Literature Review

Lymphoma staging showed that 26/35 (74%) patients were stage 3 or 4. Thirteen out of 39 patients (33%) had “B symptoms” such as night sweats, fever, and weight loss. All patients but 1 had nodal lymphoma involvement, including hilar and/or mediastinal lymphadenopathy in 12/24 (50%) patients who had an available CT-scan at lymphoma diagnosis. No patient had interstitial lung infiltration on thoracic CT. The γglobulin level was normal when available (n = 6) at lymphoma diagnosis, and except for 1 patient with hypercalcemia, calcium levels were normal (n = 7).

### Treatment of Lymphoma and Response to Therapy

Thirty-seven patients (95%) received chemotherapy, and 2 patients with HL were treated with radiotherapy alone. In addition to chemotherapy, 13 patients (33%) received Rituximab prior to sarcoidosis onset. Radiotherapy was used for 12 patients before sarcoidosis onset (31%), including patients with HL (n = 8), B-cell NHL (B-NHL, n = 3), and T-cell lymphoma (n = 1). Six patients (15%) had intensification treatment followed by autologous hematopoietic stem cell transplant (HSCT) for B-NHL, whereas 2 patients had total body irradiation followed by autologous HSCT (n = 1) or allogenic bone marrow transplantation (BMT, n = 1). One patient also underwent total gastrectomy for a malignant gastric lymphoma (DLBCL) in addition to chemotherapy.^23^

Thirty-six patients (92%) had complete response to treatment, and 3 patients (8%) had a PR followed by relapse a few months later requiring second-line therapy. Overall, 10 patients (26%) had relapse of lymphoma. In addition, a patient treated with chlorambucil for follicular NHL exhibited high-grade transformation after sarcoidosis onset.^10^ All relapsing patients were treated with chemotherapy except for 1 patient treated with rituximab alone for nodular lymphocytic predominance or type I HL (Poppema–Lennert paragranuloma) relapse. Five patients had intensification treatment followed by HSCT for HL relapse. Overall, 10 patients (26%) had a HSCT prior to sarcoidosis onset.

On last evaluation, after a median follow-up of 48 months, 36 patients (92%) had a maintained CR. Two patients had active lymphoma and 1 patient had died after allogenic BMT but without any evidence of malignancy at autopsy.

### Characteristics of Sarcoidosis

Main characteristics of sarcoidosis diagnosis, treatment, and outcome are summarized in Tables 1 Tables 2   and Table 3. Median age at sarcoidosis onset was 49 years (range, 23–71 years), and patients were predominantly women (women to men ratio of 2:1).

The median time between lymphoma diagnosis and the sarcoidosis diagnosis was 18 months (range, 1–390 months). When considering the last lymphoma relapse, the median interval until sarcoidosis onset was only 12 months (range, 1–390 months). The lymphoma was in CR at the presentation of sarcoidosis in all but 2 patients. Most patients developed sarcoidosis after the end of chemotherapy.

Sarcoidosis was identified after a systematic CT-scan or ^18^FDG-PET/CT follow-up evaluation in 18/34 patients (53%), whereas it was identified because of clinical manifestations in 16 patients (47%). Thoracic involvement was noted in 37 patients, with either hilar or mediastinal lymph nodes or with pulmonary involvement. At least 29 patients (74%) had mediastinal or hilar lymph node enlargement on chest x-ray, CT-scan, or ^18^FDG-PET/CT examination. Other manifestations involved the skin in 8 patients (20%) (including erythema nodosum in 3 patients, ichthyosis and panniculitis in 1 patient each), constitutional symptoms (fever, night sweats, and weight loss) in 10 patients (26%), joints in 6 patients (15%), eyes with uveitis in 3 patients (8%), spleen in 2 patients (5%), and heart, muscles, and central nervous system (myelitis and meningitis) in 1 patient each (3%).

Two patients had hypercalcemia that was severe in 1 case leading to acute renal failure. LDH level was normal at sarcoidosis onset in all but 1 patient. ACE level was elevated (>1.5 upper limit of normal) in 5 cases (13%). Two patients had increased γglobulins, and 7 patients had lymphopenia, including mild lymphopenia (1230–1460/μL) in 3 patients and severe lymphopenia (140–660/μL) in 4 patients.

Histological confirmation was obtained from lymph node biopsy in 22 patients (56%) including mediastinal lymph nodes in 17 patients (44%), transbronchial lung biopsy in 5 patients (13%), skin biopsy in 5 patients (13%), open lung biopsy in 4 patients (10%), bone marrow biopsy in 3 patients (8%), minor salivary gland biopsy in 2 patients (5%), and bronchial wall biopsy, lacrimal gland biopsy, and muscle biopsy in 1 case each (3%) (Figure 1).

### Treatment of Sarcoidosis and Response to Therapy

16 patients (41%) received first-line treatment for sarcoidosis, including oral corticosteroids in 14 patients at a median dose of 40 mg/d (range, 20–60 mg/d), and hydroxychloroquine (HCQ) associated with nonsteroidal anti-inflammatory drugs (NSAID) for arthralgia in 1 case. One patient received only inhaled corticosteroids.

**FIGURE 2:**
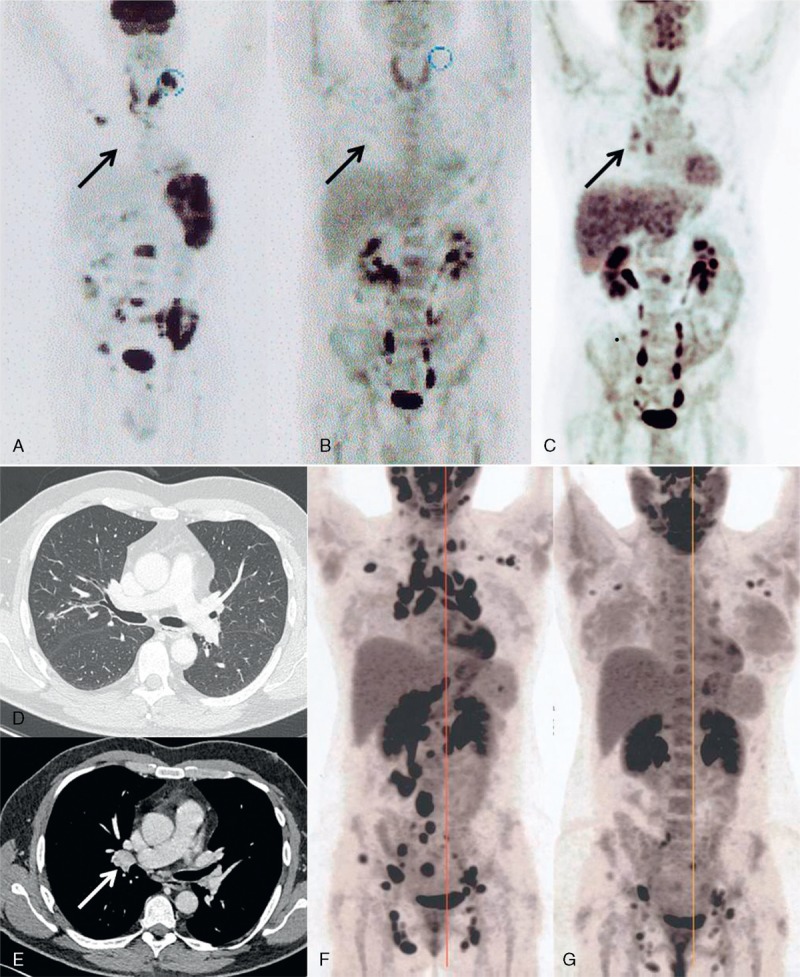
Radiological evolution of lymphoma and sarcoidosis. Positron Emission Tomography/Computerized Tomography (^18^FDG-PET/CT) of patient 1 at lymphoma diagnosis (A), lymphoma remission after diagnostic splenectomy and chemotherapy (B) and at sarcoidosis diagnosis 2 months later (C). Black arrow indicates the occurrence of moderately hypermetabolic (SUVmax = 3.5) hilar lymphadenopathy at sarcoidosis onset. CT scan at sarcoidosis diagnosis of patient 1 (D and E) shows micronodules in the middle lobe (D) and a 19 mm hilar enlarged lymph node (white arrow, panel E). Panel F and G are ^18^FDG-PET/CT evaluations of patient 2 at sarcoidosis diagnosis (F) and at last follow-up, 6 months after introduction of steroid treatment (panel G) showing a very good PR. PR = partial response, SUV = standardized uptake value.

Overall response to the treatment was good; 12/16 (75%) patients experienced clinical CR. Four patients were classified as nonresponders but the assessment of response to therapy was lacking for 3 of these patients. Six patients had radiological CR, 3 had a PR, and 1 had no radiological response (radiological response was not available for 6 patients) (Figure 2). The median duration of corticosteroid treatment was 5.25 months (range, 1–10 months).

Only 2 patients required second-line treatment after sarcoidosis relapse, 4 and 46 months after the first disease flare, respectively. One patient received cyclophosphamide and then methotrexate for severe neurological involvement (myelitis and meningitis), whereas the other received azathioprine for cardiac involvement.

After a median follow-up of 16 months after sarcoidosis onset, 32/35 patients (91%) had no clinical manifestations of sarcoidosis, whereas 15/25 patients (60%) had no radiological evidence of sarcoidosis and 4/25 (16%) had a radiological PR. Of the 3 patients with persisting clinical symptoms of sarcoidosis, 1 had myalgia and asthenia, 1 had arthralgia, and 1 patient died because of pulmonary fibrosis shortly after an allogenic HSCT without any evidence of sarcoidosis or malignancy at autopsy. Ten patients still had radiological findings of sarcoidosis on last follow-up, consisting of lymph node enlargement for most patients.

## DISCUSSION

The sarcoidosis-lymphoma syndrome described by Brincker^3,31^ was established on the basis of 2 different studies. In a Danish registry of patients with respiratory sarcoidosis, lymphoma occurred 11.5 times more frequently than expected in the general population.^31^ Second, Brincker^3^ reviewed 46 cases of lymphoma occurring in patients with sarcoidosis. He reported only 2 cases in which lymphoma preceded sarcoidosis with an additional 2 cases in a subsequent study.^24^ The evidence of the sarcoidosis-lymphoma syndrome relied on several findings. First, lymphoma occurred almost always after sarcoidosis with a short median interval of 24 months. Second, patients with the sarcoidosis-lymphoma syndrome were significantly older (median age, 41 years) by 10 years than unselected patients with sarcoidosis.^3^ Third, HL occurred more frequently than expected from epidemiological data. Overall, there were 5.5 times more cases of lymphoma observed in patients with sarcoidosis than in the general population. In the present study, we describe a similar association of sarcoidosis and lymphoma, with the particularity of sarcoidosis following lymphoma.

Initially, cases of sarcoidosis following lymphoma were disregarded by Brincker^3^ as being a random association. In the present work, we provide some findings that raise the question of a nonfortuitous association. As in Brincker’s study,^3^ we report that patients’ median age at sarcoidosis diagnosis was significantly older than the median age in unselected sarcoidosis patients (49 years in our study vs 31 years in unselected sarcoidosis patients).^32^ Strikingly, patients with sarcoidosis occurring after lymphoma are even older than patients with the sarcoidosis-lymphoma syndrome. The time interval between lymphoma and sarcoidosis was short, with a median delay of 18 months. This interval is even shorter than the 24 months first described by Brincker^3,31^ in the Danish registry or than the 95 months he later reported. Furthermore, if lymphoma relapse is taken into account, the median delay until sarcoidosis onset fell to 12 months, which supports a causal relationship. Such a short interval could also imply an undiagnosed preexisting sarcoidosis, but sarcoidosis followed lymphoma or lymphoma relapse by <6 months for only 2 patients. Alternatively, it is possible that a granulomatous reaction concomitant with the lymphoma was in fact initially present. Besides lymph node involvement, some patients later developed systemic sarcoidosis with general symptoms and skin or eye involvement, which are not usual features of sarcoid-reaction accompanying lymphoma.^5^ Finally, differentiating sarcoidosis and sarcoid-like reactions may have been difficult for some patients, in particular those with few or no clinical symptoms. Sarcoid-like reactions refer to the development of noncaseating granulomas in patients who do not fulfill the criteria for systemic sarcoidosis, usually occurring most commonly in oncology patients in the lymph nodes draining a malignant tumor. Our findings that mediastinal or hilar lymph node enlargement was present in at least 74% of patients at sarcoidosis onset, including patients who initially did not have lymphomatous mediastinal lymph nodes, strongly argue for diagnosis of sarcoidosis rather than sarcoid-like reactions.

Sarcoidosis developed shortly after partial or complete hematological response in most patients. It might be argued that the development of sarcoidosis is be related to an excessive immune response against the lymphoma cells. This immunological reaction seems to be correlated with a good prognosis because 92% of patients had a hematological CR to chemotherapy, 8% a PR, and 92% were in sustained hematological remission on last evaluation after a median follow-up of 48 months. Although the lymphoma relapsed in 10 patients, only 3 of them did so after sarcoidosis onset. This is consistent with previous findings that sarcoid-like reactions are associated with a better prognosis in patients with HL.^33,34^ Some authors previously proposed that granulomatous inflammation in the sarcoid-like reaction can be thought of as an immunological hypersensitivity reaction to tumor-derived antigens.^7^ However, because all patients received chemotherapy, radiotherapy, or rituximab, sarcoidosis could also be a reaction to such treatment. However, patients received a very wide variety of different chemotherapy; radiotherapy was used for only 31% of patients, and only 13 patients (33%) received rituximab. Moreover, some patients treated with radiotherapy presented with sarcoid reaction outside the radiotherapy field, had peripheral skin lesions, uveitis, or constitutional symptoms. Some treatments are known to trigger sarcoidosis such as interferon-α^35^ or anti-tumor necrosis factor α,^36^ but patients receiving those treatments were excluded from our study. In addition, radiotherapy and bleomycin, which are frequently used for the treatment of HL can induce lung fibrosis but not granulomatous reactions. Finally, it would be unlikely for a patient with preexisting sarcoidosis to have a disease flare after chemotherapy. Indeed, chemotherapy is very often accompanied by corticosteroids, the major treatment of sarcoidosis.

The main clinicoradiological presentation of sarcoidosis following lymphoma was the unexpected occurrence of hypermetabolic mediastinal or hilar lymphadenopathy following CR after chemotherapy or radiotherapy, frequently in asymptomatic patients. Constitutional symptoms, skin involvement, or uveitis were the most frequently associated symptoms. Therefore, it is very important for clinicians to be aware that sarcoidosis is an alternative diagnosis to lymphoma relapse when confronted with the reoccurrence of hypermetabolic mediastinal or hilar lymphadenopathies on an ^18^FDG-PET/CT evaluation in a patient with lymphoma. In a previous study, 151 patients with mediastinal lymphoma (57 HL and 94 aggressive NHL) were followed-up with an ^18^FDG-PET/CT evaluation. Out of the 30 patients who had a suspected relapse on the ^18^FDG-PET/CT and underwent systematic biopsy for confirmation, only 17 (57%) had confirmed histological relapse, 9 had benign fibrosis, 1 had an unrelated neoplastic condition, and 3 patients (10%) had sarcoid-like granulomatosis (not included in the current study for lack of specific data).^8^ Another study evaluated the prevalence of sarcoid-like reaction to malignancy detected by using ^18^FDG-PET/CT assessment. Sarcoid-like reaction was initially suspected in 23/2048 (1.1%) ^18^FDG-PET/CT evaluation and confirmed in 13 patients (0.6%), including a patient with a suspected HL relapse (not included in the current study for lack of specific data).^28^ In a third study, out of 49 suspected lymphoma relapse on ^18^FDG-PET/CT follow-up evaluation of 103 patients, 7 patients had alternative diagnosis with 1 case of sarcoidosis.^20^ Sarcoid reactions following a lymphoma might be mistaken on CT-scan or ^18^FDG-PET/CT evaluation for lymphoma relapse. This emphasizes the importance of systematic histological confirmation of lymphoma relapse to avoid unnecessary and potentially harmful intensification treatments.

The majority of cases of sarcoidosis occurring after lymphoma seem to be of mild intensity or even self-healing. Indeed, only 16 patients (41%) required treatment and 14 (36%) oral corticosteroids. Only 2 patients required immunosuppressive therapy for severe manifestations with probable cardiac involvement in 1 case and central nervous system involvement in the other. Even if only 60% of patients (15/25) had a complete radiological response on last evaluation, overall clinical response was excellent with 91% of patients being in complete clinical response. Several patients were asymptomatic at sarcoidosis presentation, therefore, it is possible that the CT-scan or ^18^FDG-PET/CT evaluation in lymphoma follow-up might result in overdiagnosis of cases of self-healing sarcoidosis that would otherwise have gone unnoticed. Therefore, sarcoidosis following lymphoma could be a fortuitous association with sarcoidosis in asymptomatic patients being simply overdiagnosed because of the radiological follow-up of patients with lymphoma. Because at the opposite we also report cases of genuine sarcoidosis following lymphoma, we can make the assumption that self-healing cases of sarcoidosis accompany the immune response to lymphoma and are underdiagnosed.

This study was not designed to determine the causal relationship between sarcoidosis and lymphoma. Indeed, it is a retrospective study combining 14 cases for which we recorded the data from patients’ medical records using a standardized form, and 25 patients reported in the literature for whom we had only limited data available. Therefore, a larger study assessing prospectively the occurrence of sarcoidosis in patients with and without lymphoma would be needed to conclude definitively that there is a nonfortuitous association.

In conclusion, sarcoidosis may occur after lymphoma diagnosis, and this association seems to be nonfortuitous. Patients with sarcoidosis following lymphoma are older than unselected sarcoidosis patients, have a short interval between lymphoma and sarcoidosis presentation, and frequently have a mild form of sarcoidosis. These patients may have an aberrant immunological reaction accompanying the immune response to the lymphoma cells, but the causal relationship between lymphoma and sarcoidosis remains to be determined by further epidemiological studies. Finally, we emphasize the necessity to rely on histological confirmation of a lymphoma relapse if suspected on ^18^FDG-PET/CT examination.
